# Curious Cases of Atropia Poisoning

**Published:** 1896-01

**Authors:** N. C. Mitra

**Affiliations:** Bengal, India, Assistant Surgeon Ranchi Municipal Hospital


### CURIOUS CASES OF ATROPIA POISONING.

BY N. C. MITRA, M.A., M.B., BENGAL, INDIA,

Assistant Surgeon Ranchi Municipal Hospital.

Three cases of atropia poisoning recently came under my treatment, all caused by the instillation of atropia drops into the eye.

1. A child aged two and a half years had an attack of acute iritis. I ordered atropia drops (gr. i. to ℥ i.). The drops had been used two or three times. At night I was sent for in haste to see the patient who was reported to be delirious. On my arrival I found the child very restless, talking deliriously, with staggering gait, some times humming songs, at others laughing. There was little difficulty in diagnosing the case as one of atropia poisoning. I stopped the eye drops, and prescribed strong coffee. The child was not allowed to sleep. There were no other untoward symptoms, and the child was quite well the next morning.

The next case was that of an old man. He was suffering from cataract of the right eye. One drop of atropia solution (gr. $\frac{1}{2}$ to ℥ i.) was instilled into the eye to dilate the pupil. After walking over a mile on his way home, he felt that he was not in his right senses, his gait was staggering, with dryness of the throat, and he had no knowledge when he reached home.

The third case was of a hospital patient admitted for cataract. Atropia drops (gr. $\frac{1}{2}$ to ℥ i.) were used. In a few minutes he had all the symptoms of atropia poisoning.

In all these cases, the poisoning was due either to the passage of the lotion through the lachrymal duct into the throat, or to its absorption by the conjunctiva.

### OXALATE OF CERIUM AS A GASTRIC SEDATIVE.

As a gastric sedative in the vomiting of pregnancy, it stands high. I have seen admirable effects from its use. In the case of a pregnant female who aborted in her previous pregnacy from obstinate vomiting, the vomiting was so troublesome that she could not retain anything in her stomach. I tried vin ipecac, tinct. nux, liquor arsenicalis, hydrocyanic acid, bismuth, and others, but without any permanent effect. It was at last, that I used oxalate of cerium, and after using it for three days the vomiting became less. Continuing it longer, it ceased once for all, and the patient was delivered of a child at full time.

In a case of vomiting after an attack of cholera, the patient had such constant retching and vomiting that the stomach rejected any food, liquid or solid, and in consequence, his case was assuming a graver aspect, when I prescribed oxalate of cerium in 5 grains, taken every six hours. The effects were marvellous, the vomiting stopped, the patient took liquid food and retained it, and was on the way to recovery.

In hiccough, an old man past fifty had been subject to this complaint for a long time. It used to persist for a long period, and was much distressing to the patient. In former attacks I gave him hydrocyanic acid, bismuth, chloroform, and it took nearly a week to control it. A few days ago he called on me suffering from a recurrence of his old complaint. The hiccough was so harrassing that the patient had to pass many a sleepless night. I gave him three powders of cerii oxalas 5 grains each. The next morning he reported himself to be infinitely better. He was given three more powders, and on inquiry I learned that he is quite free from this complaint.

---

BETWEEN theatrical bill-board posters and anatomical cigarette cards, the literature on lost manhood so generally distributed by the "retired missionary from India," and the diurnal pelvic patent medicine advertisement, the young person of the present day probably knows much more about himself and others than his great-grandfather did when he came to give up the ghost.—*Times and Register*.

